# More Evils of German and Austrian Clinics

**Published:** 1919-09-06

**Authors:** 


### GRADUATE TEACHING.

### More Evils of German and Austrian Clinics.

For decades of years it has been the practice for advanced students anxious to learn and master all new methods of treatment, to look to German and Austrian universities as centres where they must spend a year or so in study, in order to fully equip themselves.

In 1912, before the war, we published a guarded account of the evils and consequence of a rigid discipline, and the absence of an exhibition of due regard to the feelings and rights of the patients under treatment, both in Berlin and Vienna, as in many other foreign hospitals. The efficiency of any system, however admirable, must depend largely upon the spirit which dominates the individual men and women who bear the chief responsibility for its conduct. In Germany there was markedly before the war a much less keen sense of tenderness and humanity towards the sick, and, indeed, towards each other, than we, or the majority of us in Great Britain or the United States, habitually practise. The demands of science entail discipline on the patients and the speedy carrying out of whatever has to be done in connection with their treatment. The enforcement of discipline, which is the keynote of the whole of the system of relations between the father and his family, the chief and his subordinates, emphasises the responsibility which attaches to the individual heads of every department in the German and Austrian hospital. In Germany it is remarkable to notice the extraordinary deference which, up to the time of the war, was displayed to the professor by all the workers in his laboratories. All this subordination to the chief was made even more striking and complete by the adoption often of an identical attitude by the chief of the professor's whole staff. In such circumstances treatment of a patient, whether it be in the operation theatre or in any other department of the hospital, must largely depend upon the attitude of mind and the character and the principles which underlie the conduct of each chief of department, throughout a great establishment, where there were sometimes as many as 2,000 patients, and the huge staff required to minister to their necessities. The apprehension of these conditions and the observation of kindly forethought by some professors made us realise the actual position of each patient. The German hospitals, as a class, have not contained anyone, as in the voluntary hospitals in England and the United States, whose special privilege and duty it is to consider the interests of the patients all the time, and to minister tenderly to their comfort. Scientific treatment abroad is apt to control affairs completely, to the setting back of humanity and the infinite lessening of the humanising influence which the spirit of personal service has brought about, as witness the delightful atmosphere to be found in the best administered of our voluntary hospitals in Great Britain, and some of the most admirable hospitals in the United States of America. Graduates who have studied in the hospitals and medical centres of Germany and Austria can testify, as we can from observation, that in practice, where science comes first, and dominates the whole spirit of the administration of a great medical cure-house, there it will frequently be found that the spirit of personal service and infinite tenderness and human sympathy for the individual patient cannot, and does not, continually prevail.

Protected but not Protectors!

It has always been our assured feeling, as the result of over forty years' experience of voluntary hospitals, conducted as they are under a system which puts the interests of the patients, and not science, before every other consideration, that those who are wise will, when dangerously ill, seek treatment in a clinical hospital. We may add, however, as the result of our experience and knowledge during all these years, that no earthly consideration would induce us willingly to occupy a bed in a foreign teaching or other hospital where science was the first consideration and dominated every act and thought of those responsible for the treatment and handling of disease in the various wards. We are speaking from the mere man's point of view, but it must be infinitely worse for any woman patient in such a hospital. This danger is one to which every statesman and every thinking man and woman in Great Britain should become fully alive, in the interests of the sick and suffering who are mainly dependent upon the hospitals when ill or when stricken by accident or other sudden disablement. The hospitals are properly the protectors of the scientific worker in the field of medicine. It follows that where an iron discipline deprives the patients in a hospital of the safeguards of an independent and articulate public opinion, there the independence of every scientific worker must be zealously protected, or the patients may suffer grievous wrongs.

